# Dicarbon­yl(η^5^-cyclo­penta­dien­yl)(2,3-di­bromo­propanamine-κ*N*)iron(II) tetra­fluoridoborate

**DOI:** 10.1107/S1600536812025925

**Published:** 2012-06-16

**Authors:** Cyprian M. M’thiruaine, Holger B. Friedrich, Evans O. Changamu, Manuel A. Fernandes

**Affiliations:** a School of Chemistry, University of KwaZulu-Natal, Private Bag X54001, Durban 4000, South Africa; b Chemistry Department, Kenyatta University, PO Box 43844, Nairobi, Kenya; c School of Chemistry, University of the Witwatersrand, PO Wits, 2050 Johannesburg, South Africa

## Abstract

The title compound, [Fe(η^5^-C_5_H_5_)(NH_2_CH_2_CHBrCH_2_Br)(CO)_2_](BF_4_) contains an Fe^II^ cation with a three-legged piano-stool coordination. The NH_2_CH_2_CHBrCH_2_Br ligand contains a chiral carbon atom. The Fe—N bond length is 2.011 (3) Å and the Fe—Cp centroid distance is 1.7189 (5) Å. In the crystal, the ions are linked *via* two N—H⋯F inter­actions and a weak N—H⋯Br inter­action.

## Related literature

For the synthesis of the title compound and our previous work in this area, see: M’thiruaine *et al.* (2012*b* ▶). For related amino complexes, see: M’thiruaine *et al.* (2011*a* ▶,*b* ▶, 2012*a* ▶,*b* ▶). For piano-stool bromo­alkyl complex structures, see: Friedrich *et al.* (2001 ▶, 2004 ▶). For some applications of halogenated compounds, see: Gerebtzoff *et al.* (2004 ▶); Butler & Sandy (2009 ▶).
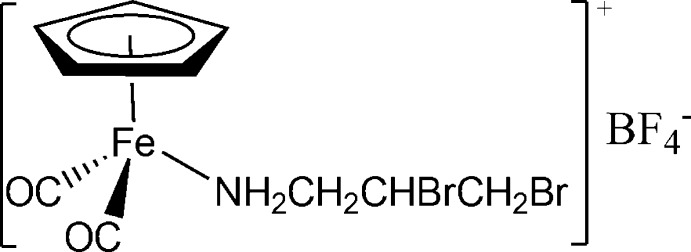


## Experimental

### 

#### Crystal data

[Fe(C_5_H_5_)(C_3_H_7_Br_2_N)(CO)_2_]·(BF_4_)
*M* *_r_* = 480.69
Monoclinic, 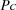

*a* = 12.9385 (4) Å
*b* = 6.7123 (2) Å
*c* = 13.2959 (4) Å
β = 138.664 (2)°
*V* = 762.65 (4) Å^3^
*Z* = 2
Mo *K*α radiation
μ = 6.27 mm^−1^
*T* = 173 K
0.54 × 0.48 × 0.29 mm

#### Data collection

Bruker APEXII CCD diffractometer
Absorption correction: integration (*SADABS*; Bruker, 2005 ▶) *T*_min_ = 0.133, *T*_max_ = 0.264
8733 measured reflections
2944 independent reflections
2751 reflections with *I* > 2σ(*I*)
*R*_int_ = 0.036

#### Refinement

*R*[*F* ^2^ > 2σ(*F* ^2^)] = 0.027
*wR*(*F* ^2^) = 0.076
*S* = 1.10
2944 reflections
190 parameters
2 restraints
H-atom parameters constrained
Δρ_max_ = 0.76 e Å^−3^
Δρ_min_ = −0.64 e Å^−3^
Absolute structure: (Flack, 1983 ▶)
Flack parameter: −0.003 (9)

### 

Data collection: *APEX2* (Bruker, 2005 ▶); cell refinement: *SAINT-Plus* (Bruker, 2005 ▶); data reduction: *SAINT-Plus* and *XPREP* (Bruker, 2005 ▶); program(s) used to solve structure: *SHELXS97* (Sheldrick, 2008 ▶); program(s) used to refine structure: *SHELXL97* (Sheldrick, 2008 ▶); molecular graphics: *ORTEP-3* Farrugia (1997 ▶); software used to prepare material for publication: *SHELXL97* and *PLATON* (Spek, 2009 ▶).

## Supplementary Material

Crystal structure: contains datablock(s) I, global. DOI: 10.1107/S1600536812025925/fj2567sup1.cif

Structure factors: contains datablock(s) I. DOI: 10.1107/S1600536812025925/fj2567Isup2.hkl

Additional supplementary materials:  crystallographic information; 3D view; checkCIF report

## Figures and Tables

**Table 1:** Hydrogen-bond geometry (Å, °)

*D*—H⋯*A*	*D*—H	H⋯*A*	*D*⋯*A*	*D*—H⋯*A*
N1—H1*A*⋯F4	0.92	2.15	2.951 (4)	145
N1—H1*A*⋯Br1	0.92	2.79	3.238 (3)	111
N1—H1*B*⋯F1^i^	0.92	2.06	2.935 (4)	158

Symmetry code: (i) 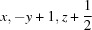.

**Fig. 1.**
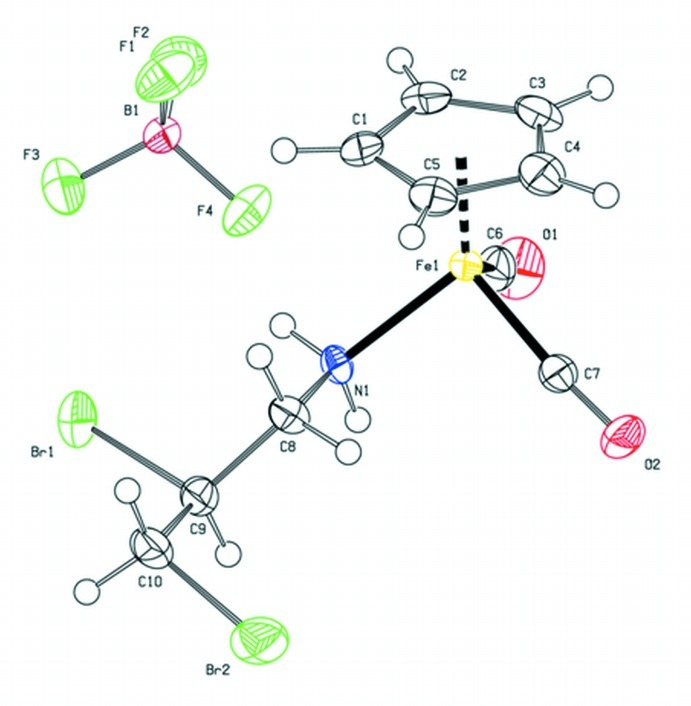
Molecular structure of the title complex with the atom labeling scheme. Ellipsoids are drawn at 50% probability level.

## supplementary crystallographic information

### Comment

Halogenated compounds are of particular importance in pharmaceuticals and agrochemicals (Butler and Sandy 2009). A significant number of drugs and drug candidates in clinical development have halogenated structures because halogenation enhances membrane binding and permeation of drugs to the target sites (Gerebtzoff *et al.*, 2004). Thus, structural determination of halogenated molecules, particularly the chiral compounds, is warranted.

The title compound (I) has been previously reported by us as the product of the bromination of the allylamino compound [Cp(CO)_2_Fe{NH_2_CH_2_CH=CH_2_}]BF_4_ (M'thiruaine *et al.*, 2012*b*). However, its molecular structure has not been previously reported. The compound is an enantiomerically pure chiral compound that crystallizes as the (*S*) enantiomer only. The unit cell of the title compound consisted of two molecular cations and two counteranions. The molecular cation displays a piano-stool geometry around the Fe^II^ ion with cyclopentadienyl coordinated to the metal centre in a pentahapto fashion, thus occupying the apical position, while two carbonyl ligands and the 2,3-dibromopropyl-1-amino ligand occupy the basal positions (Fig.1). The 2,3-dibromopropyl-1-amino ligand is coordinated to iron *via* the amino group, with an Fe—N bond length of 2.011 Å. This is within the reported Fe—N bond length range (M'thiruaine *et al.*, 2011*a*,*b*; M'thiruaine *et al.*, 2012*a*). The two Br atoms on the dibromopropylamino chain are in anti stereochemistry with a torsion angle Br1—C9—C10—Br2 =173.08 (19)^°^. The C—Br bond lengths are 1.942 (5) and 1.978 (4) Å, which are close to the 1.946 (17) and 1.971 (3) Å reported for [Cp(CO)_3_W{(CH_2_)_3_Br}] (Friedrich *et al.*, 2001) and Cp*(CO)_2_Fe{(CH_2_)_3_Br}] (Friedrich *et al.*, 2004), respectively. The molecules are linked by three hydrogen bonds namely N1—H1A—F4, N1—H1A—Br1 and N1—H1B—F1 (Table 1).

### Experimental

The title compound was prepared according to a reported procedure (M'thiruaine *et al.*, 2012*b*) and crystals were grown by layering a concentrated solution of the compound in CH_2_Cl_2_ with Et_2_O and the mixture was kept undisturbed in the dark for four days.

### Refinement

Non-hydrogen atoms were first refined isotropically followed by anisotropic refinement by full matrix least-square calculations on *F^2^* using *SHEXTL*. Hydrogen atoms were first located in the difference map then positioned geometrically and allowed to ride on their respective parent atoms.

### Crystal data

**Table:**

[Fe(C_5_H_5_)(C_3_H_7_Br_2_N)(CO)_2_]·(BF_4_)	*F*(000) = 464
*M**_r_* = 480.69	*D*_x_ = 2.093 Mg m^−^^3^
Monoclinic, *P**c*	Mo *K*α radiation, λ = 0.71073 Å
Hall symbol: P -2yc	Cell parameters from 5868 reflections
*a* = 12.9385 (4) Å	θ = 2.3–28.3°
*b* = 6.7123 (2) Å	µ = 6.27 mm^−^^1^
*c* = 13.2959 (4) Å	*T* = 173 K
β = 138.664 (2)°	Prismic, brown
*V* = 762.65 (4) Å^3^	0.54 × 0.48 × 0.29 mm
*Z* = 2	

### Data collection

**Table:**

Bruker APEXII CCD diffractometer	2944 independent reflections
Radiation source: fine-focus sealed tube	2751 reflections with *I* > 2σ(*I*)
Graphite monochromator	*R*_int_ = 0.036
φ and ω scans	θ_max_ = 28.0°, θ_min_ = 2.4°
Absorption correction: integration (*SADABS*; Bruker, 2005)	*h* = −17→16
*T*_min_ = 0.133, *T*_max_ = 0.264	*k* = −8→8
8733 measured reflections	*l* = −13→17

### Refinement

**Table:**

Refinement on *F*^2^	Secondary atom site location: difference Fourier map
Least-squares matrix: full	Hydrogen site location: inferred from neighbouring sites
*R*[*F*^2^ > 2σ(*F*^2^)] = 0.027	H-atom parameters constrained
*wR*(*F*^2^) = 0.076	*w* = 1/[σ^2^(*F*_o_^2^) + (0.0492*P*)^2^ + 0.1443*P*] where *P* = (*F*_o_^2^ + 2*F*_c_^2^)/3
*S* = 1.10	(Δ/σ)_max_ = 0.001
2944 reflections	Δρ_max_ = 0.76 e Å^−^^3^
190 parameters	Δρ_min_ = −0.64 e Å^−^^3^
2 restraints	Absolute structure: (Flack, 1983)
Primary atom site location: structure-invariant direct methods	Flack parameter: −0.003 (9)

### Special details

**Table:**

Experimental. Face indexed absorption corrections carried out with XPREP
Geometry. All e.s.d.'s (except the e.s.d. in the dihedral angle between two l.s. planes) are estimated using the full covariance matrix. The cell e.s.d.'s are taken into account individually in the estimation of e.s.d.'s in distances, angles and torsion angles; correlations between e.s.d.'s in cell parameters are only used when they are defined by crystal symmetry. An approximate (isotropic) treatment of cell e.s.d.'s is used for estimating e.s.d.'s involving l.s. planes.
Refinement. Refinement of *F*^2^ against ALL reflections. The weighted *R*-factor *wR* and goodness of fit *S* are based on *F*^2^, conventional *R*-factors *R* are based on *F*, with *F* set to zero for negative *F*^2^. The threshold expression of *F*^2^ > σ(*F*^2^) is used only for calculating *R*-factors(gt) *etc*. and is not relevant to the choice of reflections for refinement. *R*-factors based on *F*^2^ are statistically about twice as large as those based on *F*, and *R*- factors based on ALL data will be even larger.

### Fractional atomic coordinates and isotropic or equivalent isotropic displacement parameters (Å^2^)

**Table:**

	*x*	*y*	*z*	*U*_iso_*/*U*_eq_	
C1	0.2328 (5)	0.2525 (6)	0.2879 (5)	0.0312 (9)	
H1	0.3279	0.2486	0.3203	0.037*	
C2	0.1334 (5)	0.4194 (7)	0.2262 (4)	0.0341 (9)	
H2	0.1504	0.5480	0.2107	0.041*	
C3	0.0032 (5)	0.3607 (7)	0.1912 (4)	0.0344 (9)	
H3	−0.0821	0.4430	0.1482	0.041*	
C4	0.0241 (5)	0.1576 (7)	0.2321 (5)	0.0315 (8)	
H4	−0.0454	0.0791	0.2206	0.038*	
C5	0.1659 (4)	0.0924 (6)	0.2929 (4)	0.0275 (7)	
H5	0.2092	−0.0374	0.3309	0.033*	
C6	0.2019 (4)	0.5752 (6)	0.4680 (5)	0.0273 (8)	
C7	0.1693 (4)	0.2165 (6)	0.5155 (4)	0.0234 (7)	
C8	0.5160 (4)	0.1184 (5)	0.6589 (4)	0.0242 (7)	
H8A	0.4996	0.0650	0.5784	0.029*	
H8B	0.4636	0.0271	0.6685	0.029*	
C9	0.6893 (4)	0.1202 (7)	0.8079 (4)	0.0278 (8)	
H9	0.7069	0.1777	0.8892	0.033*	
C10	0.7653 (5)	−0.0805 (7)	0.8575 (5)	0.0372 (10)	
H10A	0.8802	−0.0656	0.9474	0.045*	
H10B	0.7421	−0.1421	0.7746	0.045*	
N1	0.4412 (3)	0.3170 (5)	0.6112 (3)	0.0193 (6)	
H1A	0.4852	0.3976	0.5951	0.023*	
H1B	0.4658	0.3708	0.6908	0.023*	
O1	0.1941 (5)	0.7331 (5)	0.4917 (5)	0.0451 (9)	
O2	0.1334 (3)	0.1479 (5)	0.5640 (3)	0.0363 (7)	
Fe1	0.20737 (5)	0.32704 (7)	0.42396 (5)	0.01774 (11)	
Br1	0.79524 (5)	0.28916 (9)	0.78419 (5)	0.04641 (14)	
Br2	0.68883 (6)	−0.25147 (7)	0.90878 (6)	0.04643 (14)	
B1	0.5163 (5)	0.7167 (7)	0.4432 (5)	0.0256 (8)	
F1	0.4496 (4)	0.5759 (4)	0.3313 (3)	0.0449 (6)	
F2	0.4611 (3)	0.9035 (4)	0.3751 (3)	0.0465 (7)	
F3	0.6762 (3)	0.7131 (5)	0.5531 (4)	0.0514 (7)	
F4	0.4694 (3)	0.6791 (4)	0.5072 (3)	0.0390 (6)	

### Atomic displacement parameters (Å^2^)

**Table:**

	*U*^11^	*U*^22^	*U*^33^	*U*^12^	*U*^13^	*U*^23^
C1	0.0288 (19)	0.051 (3)	0.0181 (17)	−0.0010 (17)	0.0188 (16)	−0.0024 (16)
C2	0.036 (2)	0.042 (2)	0.0227 (19)	0.0036 (17)	0.0213 (18)	0.0101 (16)
C3	0.0237 (18)	0.047 (3)	0.0176 (17)	0.0083 (18)	0.0110 (15)	0.0071 (18)
C4	0.0272 (18)	0.036 (2)	0.0233 (18)	−0.0081 (16)	0.0166 (16)	−0.0079 (16)
C5	0.0300 (17)	0.0252 (18)	0.0212 (17)	−0.0020 (15)	0.0174 (15)	−0.0047 (14)
C6	0.0273 (17)	0.0250 (19)	0.0333 (19)	0.0037 (15)	0.0239 (16)	0.0025 (16)
C7	0.0203 (16)	0.0263 (17)	0.0195 (17)	−0.0052 (14)	0.0138 (16)	−0.0037 (14)
C8	0.0209 (16)	0.0234 (18)	0.0207 (16)	0.0011 (14)	0.0134 (15)	0.0019 (15)
C9	0.0226 (17)	0.039 (2)	0.0222 (17)	0.0034 (16)	0.0171 (16)	−0.0009 (17)
C10	0.0245 (18)	0.057 (3)	0.0250 (19)	0.0150 (19)	0.0170 (17)	0.009 (2)
N1	0.0135 (12)	0.0198 (14)	0.0190 (14)	−0.0032 (10)	0.0106 (12)	−0.0026 (11)
O1	0.054 (2)	0.0288 (17)	0.062 (2)	0.0017 (14)	0.046 (2)	−0.0012 (15)
O2	0.0341 (15)	0.0485 (18)	0.0330 (15)	−0.0088 (13)	0.0272 (14)	0.0020 (13)
Fe1	0.0162 (2)	0.0201 (2)	0.0159 (2)	0.00138 (18)	0.01175 (18)	0.00273 (19)
Br1	0.0292 (2)	0.0668 (3)	0.0469 (3)	−0.0055 (2)	0.0296 (2)	0.0008 (3)
Br2	0.0550 (3)	0.0445 (3)	0.0469 (3)	0.0230 (2)	0.0404 (3)	0.0209 (2)
B1	0.0239 (19)	0.032 (2)	0.0242 (19)	0.0033 (16)	0.0190 (18)	0.0062 (17)
F1	0.0642 (17)	0.0442 (15)	0.0465 (15)	−0.0069 (14)	0.0474 (15)	−0.0093 (13)
F2	0.0506 (16)	0.0380 (15)	0.0519 (17)	0.0035 (13)	0.0387 (15)	0.0123 (13)
F3	0.0291 (13)	0.071 (2)	0.0494 (17)	0.0058 (13)	0.0282 (13)	0.0121 (15)
F4	0.0508 (16)	0.0424 (14)	0.0471 (15)	−0.0083 (12)	0.0436 (15)	−0.0048 (12)

### Geometric parameters (Å, º)

**Table:**

C1—C5	1.413 (6)	C7—Fe1	1.781 (4)
C1—C2	1.414 (6)	C8—N1	1.481 (5)
C1—Fe1	2.128 (4)	C8—C9	1.511 (5)
C1—H1	0.9500	C8—H8A	0.9900
C2—C3	1.426 (6)	C8—H8B	0.9900
C2—Fe1	2.107 (4)	C9—C10	1.497 (6)
C2—H2	0.9500	C9—Br1	1.978 (4)
C3—C4	1.417 (7)	C9—H9	1.0000
C3—Fe1	2.081 (4)	C10—Br2	1.942 (5)
C3—H3	0.9500	C10—H10A	0.9900
C4—C5	1.408 (6)	C10—H10B	0.9900
C4—Fe1	2.083 (4)	N1—Fe1	2.011 (3)
C4—H4	0.9500	N1—H1A	0.9200
C5—Fe1	2.098 (4)	N1—H1B	0.9200
C5—H5	0.9500	B1—F3	1.370 (5)
C6—O1	1.132 (5)	B1—F1	1.387 (5)
C6—Fe1	1.784 (4)	B1—F2	1.389 (5)
C7—O2	1.138 (5)	B1—F4	1.391 (5)
			
C5—C1—C2	107.9 (4)	C9—C10—H10A	109.6
C5—C1—Fe1	69.3 (2)	Br2—C10—H10A	109.6
C2—C1—Fe1	69.7 (2)	C9—C10—H10B	109.6
C5—C1—H1	126.0	Br2—C10—H10B	109.6
C2—C1—H1	126.0	H10A—C10—H10B	108.1
Fe1—C1—H1	126.5	C8—N1—Fe1	116.9 (2)
C1—C2—C3	107.9 (4)	C8—N1—H1A	108.1
C1—C2—Fe1	71.3 (2)	Fe1—N1—H1A	108.1
C3—C2—Fe1	69.1 (2)	C8—N1—H1B	108.1
C1—C2—H2	126.1	Fe1—N1—H1B	108.1
C3—C2—H2	126.1	H1A—N1—H1B	107.3
Fe1—C2—H2	125.1	C7—Fe1—C6	93.75 (18)
C4—C3—C2	107.6 (4)	C7—Fe1—N1	94.06 (15)
C4—C3—Fe1	70.2 (2)	C6—Fe1—N1	91.07 (15)
C2—C3—Fe1	71.1 (2)	C7—Fe1—C3	111.59 (17)
C4—C3—H3	126.2	C6—Fe1—C3	93.88 (19)
C2—C3—H3	126.2	N1—Fe1—C3	153.45 (15)
Fe1—C3—H3	124.2	C7—Fe1—C4	87.97 (17)
C5—C4—C3	108.1 (4)	C6—Fe1—C4	128.48 (17)
C5—C4—Fe1	70.9 (2)	N1—Fe1—C4	140.23 (15)
C3—C4—Fe1	70.0 (2)	C3—Fe1—C4	39.79 (19)
C5—C4—H4	126.0	C7—Fe1—C5	103.00 (16)
C3—C4—H4	126.0	C6—Fe1—C5	157.66 (17)
Fe1—C4—H4	124.7	N1—Fe1—C5	102.23 (14)
C4—C5—C1	108.5 (4)	C3—Fe1—C5	66.36 (17)
C4—C5—Fe1	69.8 (2)	C4—Fe1—C5	39.37 (16)
C1—C5—Fe1	71.6 (2)	C7—Fe1—C2	151.17 (16)
C4—C5—H5	125.8	C6—Fe1—C2	92.41 (19)
C1—C5—H5	125.8	N1—Fe1—C2	113.97 (14)
Fe1—C5—H5	124.5	C3—Fe1—C2	39.82 (16)
O1—C6—Fe1	178.0 (4)	C4—Fe1—C2	66.40 (17)
O2—C7—Fe1	174.9 (3)	C5—Fe1—C2	65.86 (17)
N1—C8—C9	113.8 (3)	C7—Fe1—C1	141.39 (18)
N1—C8—H8A	108.8	C6—Fe1—C1	124.63 (17)
C9—C8—H8A	108.8	N1—Fe1—C1	89.72 (14)
N1—C8—H8B	108.8	C3—Fe1—C1	66.12 (17)
C9—C8—H8B	108.8	C4—Fe1—C1	65.86 (16)
H8A—C8—H8B	107.7	C5—Fe1—C1	39.06 (16)
C10—C9—C8	114.1 (3)	C2—Fe1—C1	39.00 (17)
C10—C9—Br1	106.4 (5)	F3—B1—F1	110.9 (4)
C8—C9—Br1	108.7 (3)	F3—B1—F2	109.7 (4)
C10—C9—H9	109.2	F1—B1—F2	108.4 (3)
C8—C9—H9	109.2	F3—B1—F4	110.3 (4)
Br1—C9—H9	109.2	F1—B1—F4	109.1 (3)
C9—C10—Br2	110.3 (3)	F2—B1—F4	108.4 (3)
			
C5—C1—C2—C3	−0.7 (4)	C3—C4—Fe1—N1	−137.9 (3)
Fe1—C1—C2—C3	−59.7 (3)	C5—C4—Fe1—C3	118.4 (3)
C5—C1—C2—Fe1	59.0 (3)	C3—C4—Fe1—C5	−118.4 (3)
C1—C2—C3—C4	0.1 (5)	C5—C4—Fe1—C2	80.1 (3)
Fe1—C2—C3—C4	−61.0 (3)	C3—C4—Fe1—C2	−38.3 (2)
C1—C2—C3—Fe1	61.1 (3)	C5—C4—Fe1—C1	37.3 (2)
C2—C3—C4—C5	0.6 (5)	C3—C4—Fe1—C1	−81.1 (3)
Fe1—C3—C4—C5	−61.0 (3)	C4—C5—Fe1—C7	70.2 (3)
C2—C3—C4—Fe1	61.6 (3)	C1—C5—Fe1—C7	−171.2 (2)
C3—C4—C5—C1	−1.0 (5)	C4—C5—Fe1—C6	−67.4 (5)
Fe1—C4—C5—C1	−61.5 (3)	C1—C5—Fe1—C6	51.2 (5)
C3—C4—C5—Fe1	60.4 (3)	C4—C5—Fe1—N1	167.4 (2)
C2—C1—C5—C4	1.1 (4)	C1—C5—Fe1—N1	−74.0 (2)
Fe1—C1—C5—C4	60.3 (3)	C4—C5—Fe1—C3	−37.9 (3)
C2—C1—C5—Fe1	−59.2 (3)	C1—C5—Fe1—C3	80.7 (3)
N1—C8—C9—C10	179.4 (3)	C1—C5—Fe1—C4	118.6 (3)
N1—C8—C9—Br1	−62.1 (3)	C4—C5—Fe1—C2	−81.6 (3)
C8—C9—C10—Br2	−67.2 (4)	C1—C5—Fe1—C2	37.0 (2)
Br1—C9—C10—Br2	173.08 (19)	C4—C5—Fe1—C1	−118.6 (3)
C9—C8—N1—Fe1	−174.8 (2)	C1—C2—Fe1—C7	−109.4 (4)
C8—N1—Fe1—C7	72.1 (3)	C3—C2—Fe1—C7	9.1 (5)
C8—N1—Fe1—C6	166.0 (3)	C1—C2—Fe1—C6	148.4 (3)
C8—N1—Fe1—C3	−93.2 (4)	C3—C2—Fe1—C6	−93.2 (3)
C8—N1—Fe1—C4	−19.6 (4)	C1—C2—Fe1—N1	56.1 (3)
C8—N1—Fe1—C5	−32.1 (3)	C3—C2—Fe1—N1	174.6 (2)
C8—N1—Fe1—C2	−100.9 (3)	C1—C2—Fe1—C3	−118.5 (4)
C8—N1—Fe1—C1	−69.4 (3)	C1—C2—Fe1—C4	−80.2 (3)
C4—C3—Fe1—C7	−57.7 (3)	C3—C2—Fe1—C4	38.2 (3)
C2—C3—Fe1—C7	−175.3 (3)	C1—C2—Fe1—C5	−37.0 (2)
C4—C3—Fe1—C6	−153.3 (3)	C3—C2—Fe1—C5	81.5 (3)
C2—C3—Fe1—C6	89.1 (3)	C3—C2—Fe1—C1	118.5 (4)
C4—C3—Fe1—N1	106.5 (4)	C5—C1—Fe1—C7	13.8 (4)
C2—C3—Fe1—N1	−11.1 (5)	C2—C1—Fe1—C7	133.2 (3)
C2—C3—Fe1—C4	−117.6 (4)	C5—C1—Fe1—C6	−158.9 (2)
C4—C3—Fe1—C5	37.5 (2)	C2—C1—Fe1—C6	−39.5 (3)
C2—C3—Fe1—C5	−80.1 (3)	C5—C1—Fe1—N1	110.0 (2)
C4—C3—Fe1—C2	117.6 (4)	C2—C1—Fe1—N1	−130.6 (3)
C4—C3—Fe1—C1	80.4 (3)	C5—C1—Fe1—C3	−81.4 (3)
C2—C3—Fe1—C1	−37.2 (3)	C2—C1—Fe1—C3	38.0 (3)
C5—C4—Fe1—C7	−113.5 (3)	C5—C1—Fe1—C4	−37.6 (2)
C3—C4—Fe1—C7	128.2 (3)	C2—C1—Fe1—C4	81.7 (3)
C5—C4—Fe1—C6	153.4 (2)	C2—C1—Fe1—C5	119.4 (4)
C3—C4—Fe1—C6	35.0 (3)	C5—C1—Fe1—C2	−119.4 (4)
C5—C4—Fe1—N1	−19.5 (4)		

### Hydrogen-bond geometry (Å, º)

**Table:**

*D*—H···*A*	*D*—H	H···*A*	*D*···*A*	*D*—H···*A*
N1—H1*A*···F4	0.92	2.15	2.951 (4)	145
N1—H1*A*···Br1	0.92	2.79	3.238 (3)	111
N1—H1*B*···F1^i^	0.92	2.06	2.935 (4)	158

Symmetry code: (i) *x*, −*y*+1, *z*+1/2.
